# Diffusion model based OCT to OCTA translation

**DOI:** 10.3389/fmed.2025.1655453

**Published:** 2025-11-28

**Authors:** Rashadul Hasan Badhon, Atalie Carina Thompson, Jennifer I. Lim, Theodore Leng, Minhaj Nur Alam

**Affiliations:** 1 Department of Electrical and Computer Engineering, University of North Carolina at Charlotte, Charlotte, NC, United States; 2 Department of Surgical Ophthalmology, Atrium-Health Wake Forest Baptist, Winston-Salem, NC, United States; 3 Department of Ophthalmology and Visual Science, University of Illinois at Chicago, Chicago, IL, United States; 4 Department of Ophthalmology, Stanford University School of Medicine, Stanford, CA, United States

**Keywords:** diffusion model, BBDM, translation, OCT, OCTA, GaN, vascular features

## Abstract

**Introduction:**

This study introduces a conditional diffusion-based approach (Brown Bridge diffusion model, BBDM) for translating optical coherence tomography (OCT) images into OCT Angiography (OCTA).

**Methods:**

Traditional generative adversarial networks (GANs) often face limitations in generalization and structural fidelity due to adversarial loss and one-to-one mappings. In contrast, BBDM employs a bidirectional stochastic process that transitions directly between OCT and OCTA without intermediate conditioning, improving robustness, generalizability and structural consistency. The model was implemented in the latent space of VQGAN, trained on the OCT500 dataset and evaluated on an independent clinical dataset from the University of Illinois at Chicago (UIC) comprising diabetic retinopathy patients with varying severity.

**Results:**

Quantitative vascular features-blood vessel density (BVD), caliber (BVC), tortuosity (BVT) and vessel perimeter index (VPI) along with image-quality metrics such as structural similarity index (SSIM), Fréchet inception distance (FID), and perceptual contrast quality index (PCQI) were used for evaluation. BBDM achieved higher SSIM and PCQI scores in larger field-of-view scans, indicating improved structural preservation and perceptual fidelity compared to GAN. Although it slightly underperformed in FID and showed variability in vascular features, BBDM maintained anatomical trends consistent with ground-truth OCTA. Moreover, it reliably preserved clinically relevant features such as BVC, BVT, and VPI. Despite minor feature-level deviations, BBDM offers advantages in computational simplicity, training stability and reduced hallucinations.

**Conclusion:**

This work presents the first diffusion-based framework for OCT-to-OCTA translation and demonstrates that BBDM can generate clinically meaningful OCTA from standard OCT, supporting more accessible and cost-effective retinal disease diagnostics.

## 1 Introduction

Optical coherence tomography (OCT) is a non-invasive, high-resolution imaging technique, utilizing the principles of low-coherence interferometry, originally developed for high-resolution ranging and the characterization of optoelectronic components (1). The first biomedical application of this was in measuring eye length (2) and *in vivo* retinal imaging demonstrated in 1993 (3, 4). Early studies in 1995 provided the first demonstration of OCT imaging of the normal retina (5) and of macular pathology (6). Due to improved sensitivity, acquisition speed and resolution, OCT has been rapidly and widely adopted in ophthalmology (7) aiding in the detection and monitoring of various retinal pathologies including diabetic retinopathy (DR), age-related macular degeneration (AMD) and glaucoma that cannot be obtained by any other non-invasive diagnostic technique (8–10).

Despite the merits of OCT, the low contrast of structural OCT between blood vessels and static tissue makes it difficult to detect any vascular changes crucial to the retinopathies (11). Built on the principle of OCT, OCT Angiography (OCTA) was developed to capture both structural and dynamic vascular blood flow information (12, 13). OCTA can provide high resolution images of retinal vasculature, enabling quantifying retinal characteristics as well as reliable detection of retinal pathologies: AMD, DR, Glaucoma and other retinal diseases (14–33). However, OCTA is yet to be adopted widely due to additional requirements of hardware and software thereby imposing a significant financial strain on both clinics and patients (34, 35). As a result, only a limited number of hospitals and retinal clinics routinely use OCTA for daily ophthalmic check-ups. Another drawback of OCTA lies in the image acquisition process, which is time-consuming and requires repetitive scanning of the retina. This makes data collection challenging ultimately compromising the quality of OCTA images.

A possible and easy to implement solution can be utilizing Deep Learning (DL) algorithms to analyze OCT and generate detailed vascular images aligned with available OCT information bypassing the necessity to use separate setup namely translated OCTA (TR-OCTA). The use of TR-OCTA holds substantial promise in clinical practice by enabling non-invasive depth resolved visualization of retinal vasculature. OCTA has been shown to detect enlargement of the foveal non-flow zone and perifoveal inter capillary areas with increasing DR severity. TR-OCTA provided features could thereby support earlier detection of microvascular impairment.

Recent studies are already showing promising outcomes, producing high resolution vascular imaging (36–41). UNet based different models have been implemented attempting to leverage generative adversarial networks (GAN) starting by Lee et al. (36) however the quality of TR-OCTA was suboptimal. Another approach proposed by Li et al. (38), can produce comparatively better quality images. In our recent work (42), we adapted this method to generate OCTA images from OCT and demonstrated that we could quantify retinal vascular features [Blood Vessel Density (BVD), Blood Vessel Caliber (BVC), Blood Vessel Tortuosity (BVT), Vessel Perimeter Index (VPI)] which were comparable to that of ground truth OCTA (GT-OCTA) features for disease detection. We also compared the quality using Structural Similarity Index Measure (SSIM), Fréchet Inception Distance (FID) and patch-based contrast quality index (PCQI).

Although, image-to-image translation based on GAN shows high performance, they also suffer from lack of diverse translation due to one-to-one mapping (43). As an alternative approach, diffusion models (DM) recently have shown competitive performance as a translation algorithm, especially condition-based DM (44). A diffusion probabilistic model (45), also known as DM, in general implements the idea of forward diffusion, gradually adding noise to a data distribution leading to latent distribution then reverses the process.

Recent advances in diffusion probabilistic models (DPMs) have begun to impact medical imaging beyond retinal diagnostics, with applications in modalities such as MRI and CT. DPMs have been used for accelerated MRI reconstruction and ultra-sparse-view CT image generation, thereby improving image quality and reducing radiation dose.^1^ In brain imaging, a 3D diffusion model (Med-DDPM) was utilized to synthesize high-fidelity semantic MRI volumes, facilitating downstream segmentation and data-augmentation tasks.^2^

Despite the rapid development and performance improvement, DM based medical image translation studies are relatively few and this study is the first attempt to translate OCT to OCTA using conditional DM. We have implemented a Brownian Bridge DM (BBDM) model^3^ (43) that uses a continuous stochastic model where the diffusion process is conditioned based on the starting and ending states, regulating the noise addition in each step leading to TR-OCTA similar to the GT-OCTA. This model offers a significant advancement over conventional conditional diffusion models (CDM) for image-to-image translation. Unlike CDMs, which rely on intermediate conditional inputs and suffer from domain gaps, BBDM directly models the transformation as a stochastic Brownian Bridge process. This bidirectional diffusion approach eliminates the dependency on explicit conditional guidance at each step, ensuring a robust and efficient transition between domains. As a result, BBDM improves generalization, stability, and translation fidelity while reducing the risk of information leakage.

Moreover, BBDM incorporates an accelerated sampling process that enhances efficiency by reducing the number of inference steps without sacrificing quality. Instead of traversing every intermediate step in the reverse diffusion process, the model selectively samples key points along the diffusion trajectory, using a non-Markovian strategy. By adjusting variance at each sampled step, BBDM maintains smooth and consistent transitions, significantly cutting down computational overhead.

In this study, we present the first application of a diffusion model (BBDM) for OCT to OCTA translation, demonstrating its superior ability to generate perceptually coherent and structurally accurate vascular maps compared to traditional GAN based methods. By eliminating dependency on intermediate conditional inputs and employing a bidirectional diffusion mechanism, BBDM achieves improved generalization across datasets and retains high structural similarity, particularly in larger field-of-view scans which are evident from the TR-OCTA generated from the unseen UIC dataset during training phase. Our results highlight BBDM’s potential as a robust alternative for modality translation, paving the way for cost effective, hardware independent retinal vascular imaging in clinical practice.

## 2 Materials and methods

### 2.1 Model architecture

**Figure 1:**
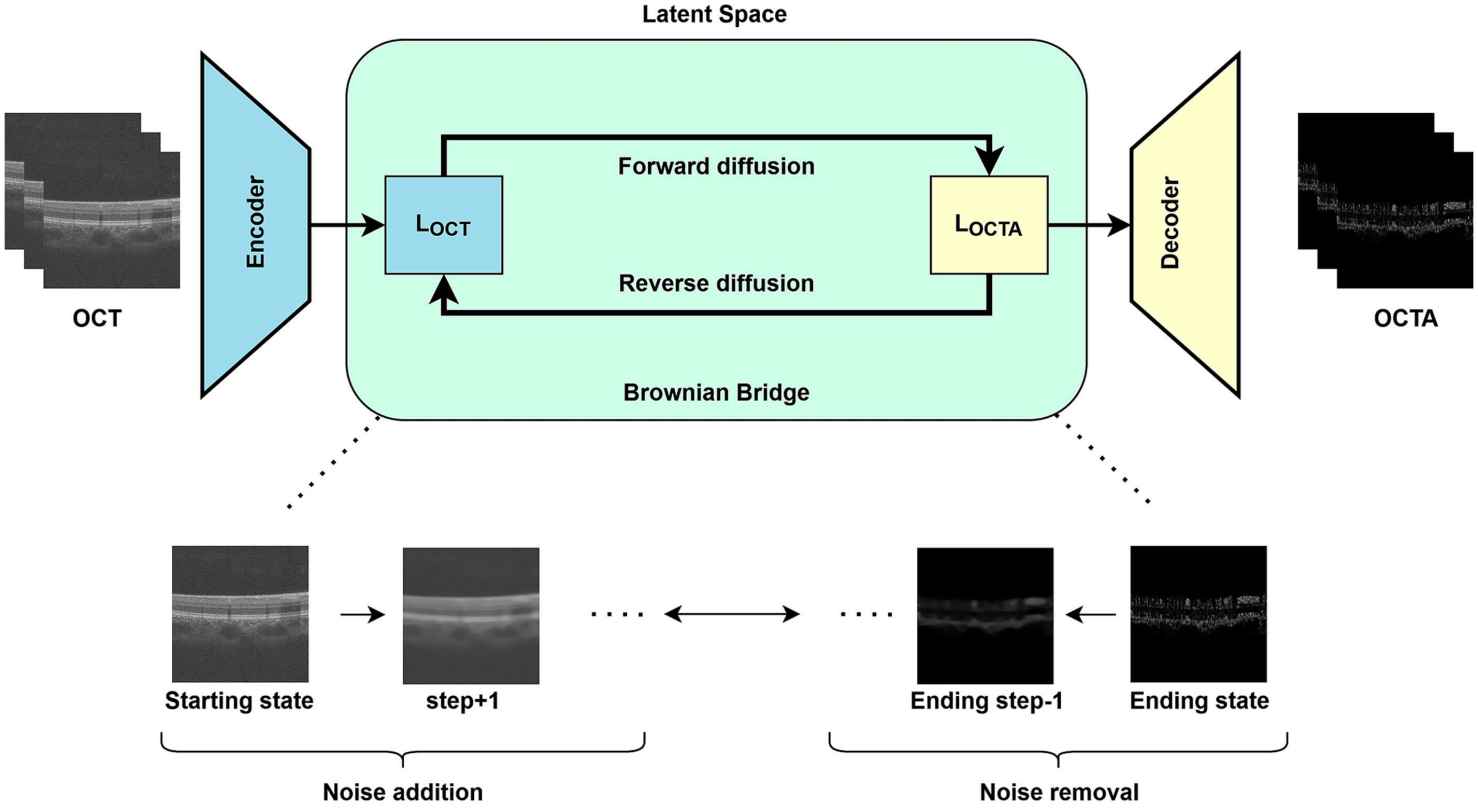
BBDM model framework.

BBDM (43) models the image-to-image translation task as a stochastic process. It builds a direct mapping between the latent representations of the source and target domains using the principles of Brownian Bridge diffusion (Figure 1). Unlike other DM which end at a standard Gaussian noise, the Brownian Bridge process concludes at the latent representation of the target domain. The core idea here is to replace the traditional conditional input mechanism with a stochastic bridging process. This eliminates dependency on intermediate conditional information at each diffusion step, making the method theoretically robust and practically effective. The proposed method is implemented in the latent space of VQGAN (46), a pre-trained model known for efficient and generalizable representation learning. We trained our BBDM model for 100 epochs using the Adam optimizer with a learning rate of 1e−4 and momentum, β_1_ = 0.9. A learning rate scheduler with a decay factor of 0.5 (patience: 10 epochs, minimum LR: 5e−7) was employed. The training was conducted with gradient accumulation enabled (accumulate_grad_batches = 2). An exponential moving average was applied to the model weights with a decay rate of 0.995, updated every 8 steps after an initial 30,000 step warm-up. The model was trained using an L1 loss, applied to the prediction of the gradient of the target image. We used a 2D UNet backbone with six input channels and three output channels. The Brownian Bridge conditioning was implemented using 1,000 diffusion steps and sampling was performed using a linear bridge schedule with 20 inference steps.

In the BBDM, the forward process involves gradually changing the image information from source domain (OCT) to target domain (OCTA). This is done through a process that slowly adds controlled amounts of random noise over several steps (stochastic representation). It starts with a clear version of the OCT image and begins to introduce noise in a smart and balanced way, mixing it more and more with the features of an OCTA image. This change is carefully guided using a variance scheduler so that it does not happen too fast or too slow as it’s designed to reach its most mixed or “noisy” state halfway through and then settle down as it gets closer to the OCTA image. By the end of this step, the image has been changed in a way that it now strongly resembles the OCTA scan, setting things up for the next phase where the noise will be removed to generate the result.

The reverse process in the BBDM reconstructs the clean latent representation of the target domain (OCTA) from the noisy intermediate state. Unlike traditional DMs that rely on conditional inputs at every step, this approach removes noise iteratively based on learned patterns, without additional external conditions. At each step of the reverse process, the model predicts the mean value of the latent representation and adjusts the noise variance to progressively refine the intermediate states toward the final clean representation. This process starts with the latent representation of the target domain and iteratively predicts and removes noise until a clean and accurate representation is obtained.

### 2.2 Dataset

We utilized two datasets for our study: the publicly available OCT500 dataset (47), which includes paired 3D OCT and OCTA volumes from 500 patients, and a second dataset comprising 445 scans of DR patients collected from UIC, containing OCT volumes and corresponding OCTA projections.

#### 2.2.1 OCT500

This dataset is divided into two subsets based on their field of view (FoV): 3 mm and 6 mm. The translation algorithm is applied separately to each subset for comparison.

The 3 mm subset consists of paired OCT and OCTA volumes from 200 patients, with a FoV of 3 mm × 2 mm × 3 mm. Each volume contains 304 slices, with a resolution of 640 × 304 pixels per slice. The corresponding OCTA projection maps have a size of 224 × 224 pixels. This subset is split into training, validation, and test sets in a 70–5–25% ratio with a *k* = 3 fold cross-validation, resulting in 140 training volumes, 10 validation volumes, and 50 test volumes.

The 6 mm subset includes paired OCT and OCTA volumes from 300 patients, with a FoV of 6 mm × 2 mm × 6 mm. Each volume contains 400 slices, with each slice measuring 640 × 400 pixels. The generated OCTA projection maps are also 224 × 224 pixels. Similar to the 3 mm set, this subset follows a 70–5–25% split, with 180 volumes for training, 20 for validation, and 100 for testing.

#### 2.2.2 UIC data and image acquisition

This study was approved by the Institutional Review Board at the University of Illinois at Chicago and followed the ethical guidelines outlined in the Declaration of Helsinki. Patients diagnosed with DR were selected from the UIC Retina Clinic. We performed a retrospective study of consecutive diabetic patients (Type II) who underwent OCTA and OCT imaging. The patients are thus representative of a university population of diabetic patients who require imaging for management of diabetic macular edema and DR. OCT/OCTA images of both eyes of every patient were collected. We excluded subjects with macular edema, previous vitreous surgery and history of other eye diseases. All participants underwent a comprehensive eye examination, including anterior segment evaluation and dilated fundus examination, performed by a retina specialist (J.I.L.). The patients were classified by severity of NPDR (mild, moderate, and severe) according to the Early Treatment Diabetic Retinopathy Study staging system. Grading was conducted by a retina specialist using slit-lamp biomicroscopy with a fundus lens. OCT/OCTA data were acquired using an ANGIOVUE spectral domain OCTA system (Optovue, Fremont, CA, USA), with a 70-kHz A-scan rate, an axial resolution of 5 μm, and a lateral resolution of 15 μm. All scans focused on the macular region with a FoV of 6 mm. The resulting OCTA images were exported using ReVue software (Optovue) and analyzed further using custom-built Python scripts with OpenCV for processing, feature extraction and classification.

The UIC dataset contains 445 OCT scans from 41 patients with different Non-Proliferative DR conditions: control, mild, moderate and severe NPDR. The scans were selected based on signal strength *Q* ≥ 5 for this study. Similar to OCT500, this dataset has both 3 mm (187 scans) and 6 mm (258 scans) scans for different stages of DR: Control, Mild, Moderate and Severe. For 3 mm FOV, we used 35 scans for Control group, 118 for Mild, 37 for Moderate and 97 for Severe. On the other hand, for 6 mm, the set included 59 for Control, 143 Mild, 69 Moderate and 123 Severe scans for comparison. 3 mm slices are of size 640px × 304px and 6 mm slices are mostly of 640px × 400px with some mixed 640px × 304px scans which are used to generate 224px × 224px OCTA slices. Some patients were listed in multiple categories therefore, scans of those patients were included in multiple categories before feature evaluation.

### 2.3 Performance metrics

We used several metrics for similarity and quality comparison. Quality metrics: SSIM, FID and PCQI. Quantitative retinal features: BVD, BVC, BVT and VPI for quality comparison. All these metrics have been elaborately discussed in Supplemental material section 1.

### 2.4 Qualitative metrics

The qualitative evaluation includes both expert-based and perceptual image quality metrics to assess the realism of the generated OCTA images. A hallucination quality score (0–10) was assigned by an expert clinician based on the presence of artificial vascular structures, where a lower score indicates fewer hallucinated vessels and a higher score reflects stronger hallucination artifacts. Additional metrics includes the SSIM, which compares the structural, luminance and contrast similarity between real and generated images; the FID, which quantifies the distance between feature distributions of real and generated images using deep neural network embeddings (lower FID implies higher image realism) and the PCQI, which evaluates perceptual contrast fidelity based on the human visual system’s sensitivity to local contrast variations.

### 2.5 Quantitative metrics

BVD Blood vessel density or vessel density (48) represents the ratio of perfused vessel area to total image area, serving as an indicator of retinal vascular health (49, 50). In OCTA imaging, BVD quantifies the extent of perfused capillaries and is sensitive to local vascular changes. BVC, another feature used for this study, calculates the ratio of vessel area to the vessel length (29) and is a critical quantitative vascular biomarker. In ophthalmic imaging, BVC provides insight into vascular adaptations or pathologies: for example, arterial narrowing may indicate systemic hypertension, while venous dilation could suggest retinal ischemia or compromised perfusion (51). BVT (18) measures the degree of curvature and directional variation along vessels, reflecting microvascular remodeling observed in diseases like sickle cell retinopathy and Fabry disease (52, 53). VPI (50) normalizes the total vessel boundary length to the image area, quantifying vascular boundary complexity associated with branching or dilation. VPI has been shown to significantly increase in retinal conditions characterized by microvascular remodeling such as non-proliferative DR and sickle cell retinopathy (SCR) where capillary dilation and branching complexity produce longer vessel boundaries relative to imaged area.

To draw the comparison between GAN model and DM we also performed the same statistical analysis based on these features to quantify the TR-OCTA. This will provide a clear distinction for the DM as a translation algorithm for medical imaging.

We performed two-tail t-test for both datasets separately for 3 mm and 6 mm. We considered the whole 3 mm or 6 mm because our datasets, especially OCT500 has mixed pathological patients causing inherent variability among the patients and across any other dataset with dissimilarities. For this reason, we reported means, standard deviations, and visualized trends across disease stages, as this reflects the clinical relevance of trend fidelity rather than inferential statistical tests. Since TR-OCTA is generated from structural OCT and not direct flow data, perfect alignment in absolute values is not expected rather we focused more on qualitative agreement, quantitative characterization and clinical interpretability. We agree that formal hypothesis testing with corrections (e.g., Bonferroni, FDR) and effect size reporting (Cohen’s d) would strengthen future work, particularly with larger, more homogeneous datasets.

## 3 Results

**Figure 2:**
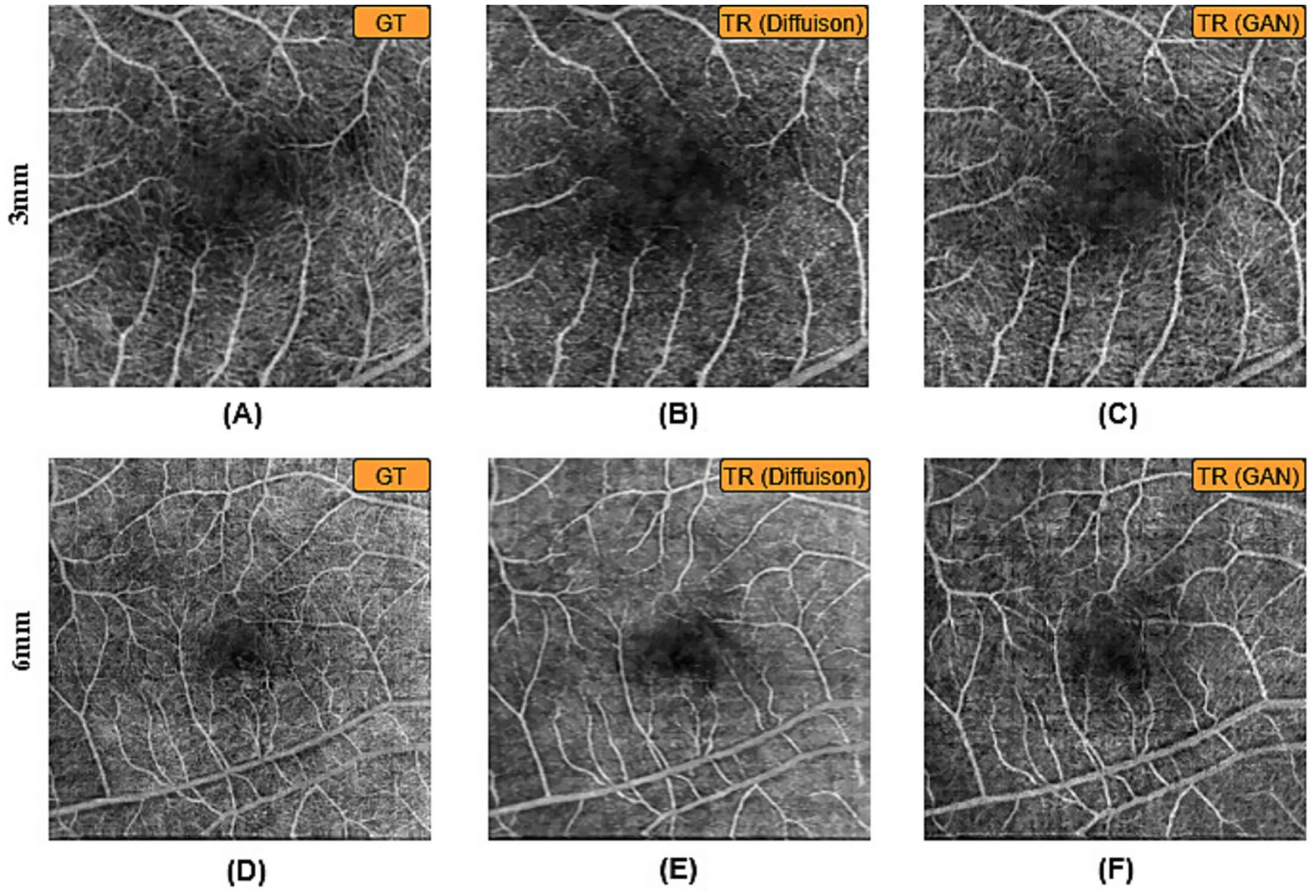
TR-OCTA generated by the diffusion (BBDM) and GAN model. **(A,D)** represent GT-OCTA and **(B,C,E,F)** show generated TR-OCTA.

The BBDM was solely trained on OCT500 and tested on UIC dataset for which we compared the quality as well as feature values with our previous work of GAN based model to observe the performance of diffusion algorithms for ophthalmic images for the first time. Due to resource as well as time restrictions, the image size was reduced to 224 × 224 for the DM as opposed to the GAN model generated slices having a size of 256 × 256. However, to ensure fair comparison, all GAN-generated images were resized to 224 × 224 prior to evaluation. Thus, all quantitative and qualitative analyses were conducted at the same resolution, avoiding bias from mismatched image sizes. Figure 2 represents the comparative quality of the DM generated OCTA from GT-OCTA.

As a part of quantitative analysis, we first considered the two-tailed T-test (*p* < 0.05) across the OCT500 and UIC datasets for both 3 mm and 6 mm. The DM demonstrated significant differences in most quantitative features across both datasets, as indicated by the extremely low *p*-values in the two-tailed t-test (Table 1). For the OCT500 3 mm, BBDM exhibited moderate differences between TR-OCTA and GT-OCTA in BVD (*p* = 0.76) however, BVC, BVT and VPI showed statistically significant differences, In the 6 mm FoV, the DM showed even stronger deviations with BVD (*p* = 0.013) and highly significant differences in BVC, BVT and VPI. When tested on UIC dataset, DM exhibited even more pronounced deviations, with extremely low *p*-values across all features. In the 3 mm dataset, BVD BVC, BVT and VPI all followed the same trend, emphasizing a larger discrepancy in vascular structure. The 6 mm dataset showed even more extreme differences, particularly for VPI, BVT, and BVC resulting in diffusion based model’s struggles to maintain accurate feature distributions in the UIC dataset. In comparison, the GAN-based model exhibited lower deviations in some cases but still showed statistically significant differences overall. For the OCT500 dataset, BVD remained non-significant at 3 mm and 6 mm, whereas BVC and VPI had highly significant differences in 6 mm. In the UIC dataset, the GAN model demonstrated statistical differences in nearly all features, particularly BVC for 3 mm and 1 6 mm, reflecting deviations in vessel connectivity across datasets.

**Table 1:** Comparison of Two tail t-test for both FoV from both OCT500 and UIC dataset.

Quantitative features	OCT500 3 mm (*p* < 0.05)	OCT500 6 mm (*p* < 0.05)	UIC 3 mm (*p* < 0.05)	UIC 6 mm (*p* < 0.05)
Diffusion model (BBDM)				
BVD	0.76	0.013	5.82 $e^{-30}$	5.61 $e^{-18}$
BVC	1.2 $e^{-4}$	3.55 $e^{-6}$	1.24 $e^{-31}$	7.41 $e^{-79}$
BVT	0.02	2.26 $e^{-9}$	1.06 $e^{-26}$	3.83 $e^{-49}$
VPI	1.97 $e^{-32}$	2.75 $e^{-43}$	3.22 $e^{-24}$	2.4 $e^{-124}$
GAN based model				
BVD	0.48	0.58	1.7 $e^{-31}$	4.91 $e^{-15}$
BVC	0.45	1.35 $e^{-52}$	7.14 $e^{-115}$	1.5 $e^{-106}$
BVT	1.1 $e^{-7}$	0.006	1.54 $e^{-41}$	6.76 $e^{-14}$
VPI	1.36 $e^{-22}$	8.26 $e^{-31}$	3.89 $e^{-5}$	0.040

While calculating SSIM (Table 2), the DM achieved an SSIM of 0.4892 for the 3 mm OCT500 dataset, which was slightly lower than the GAN-based model (0.5011). However, in the 6 mm dataset, the BBDM outperformed the GAN-based model with a higher SSIM, suggesting improved image reconstruction quality at larger FoVs. For the UIC dataset, the DM demonstrated slightly higher SSIM values across patient groups in 3 mm compared to the GAN-based model. This trend continued for 6 mm, where the DM attained an SSIM of 0.3211, outperforming the GAN-based model (0.2952). The improved SSIM in 6 mm suggests that the model preserves more structural details in larger scan areas, despite greater feature variations.

**Table 2:** Comparison of SSIM values for both OCT500 and UIC dataset.

BBDM		
SSIM	3 mm	6 mm
OCT500	0.4892 (0.299–0.57)	0.5567 (0.15–0.65)

SSIM (UIC)	Complete dataset	Control	Mild NPDR	Moderate NPDR	Severe NPDR
3 mm	0.2881 (0.11–0.41)	0.3264 (0.17–0.41)	0.2744 (0.14–0.37)	0.2912 (0.11–0.39)	0.2709 (0.11–0.39)
6 mm	0.3211 (0.09–0.42)	0.3237 (0.14–0.42)	0.3166 (0.09–0.42)	0.3148 (0.14–0.42)	0.2912 (0.12–0.41)

GAN		
SSIM	3 mm	6 mm
OCT500	0.5011 (0.29–0.60)	0.4257 (0.25–0.51)

SSIM (UIC)	Complete dataset	Control	Mild NPDR	Moderate NPDR	Severe NPDR
3 mm	0.2808 (0.13–0.39)	0.3188 (0.21–0.38)	0.2961 (0.14–0.35)	0.2914 (0.13–0.39)	0.2647 (0.13–0.39)
6 mm	0.2952 (0.12–0.38)	0.2820 (0.12–0.36)	0.2966 (0.19–0.37)	0.2920 (0.12–0.38)	0.2679 (0.12–0.37)

To further assess generative model performance, FID and PCQI scores were computed for both datasets (Table 3). For both metrics we calculate the individual slice score and take the average of all the calculated scores as well as the standard deviation to represent numerical comparison. For 3 mm OCT500, the diffusion model exhibited a much higher FID score of 110.02, indicating lower image fidelity compared to the GAN-based model (45.13). However, for the 6 mm dataset, the diffusion model improved significantly, achieving a lower FID of 66.08, while the GAN-based model had a score of 61.38. For the UIC dataset, the diffusion model displayed the highest FID values across all settings, with 222.71 for 3 mm and 153.27 for 6 mm, compared to 150.34 and 107.74 in the GAN-based model. Despite these differences, the PCQI values for the diffusion model remained high, with the 6 mm OCT500 dataset achieving 0.9984, slightly surpassing the GAN model’s 0.9978. This indicates that while FID suggests reduced fidelity, the perceptual quality of images remains intact.

**Table 3:** FID and PCQI scores for both datasets.

OCTA dataset	FID	PCQI (Mean, SD)
BBDM		
OCT500 3 mm	110.02	0.9978 ± 0.0004
OCT500 6 mm	66.08	0.9984 ± 0.0004
UIC 3 mm	222.71	0.9952 ± 0.0008
UIC 6 mm	153.27	0.9959 ± 0.00053
GAN		
OCT500 3 mm	45.13	0.9981 ± 0.0004
OCT500 6 mm	61.38	0.9978 ± 0.0005
UIC 3 mm	150.34	0.99546 (0.000737)
UIC 6 mm	107.74	0.99555 (0.000606)

We also compared the hallucination quality based on a scoring system provided by a clinician. We used random manual review of the translated OCTA projection images to ensure quality control. The manual graders provided a vascular hallucination score (0–10, lower score is better – more than five 10 × 10 patches within an OCTA image with fake or hallucinated vascular structures will constitute a score of 10). Average score was 2.3 for our algorithm, compared to 5.7–8.4 in other baseline methods (GAN based model) on a sample of 500 TR-OCTA images.

Aside from the quality comparison, we also conducted feature analysis for BVD, BVC, BVT and VPI for both datasets (Table 4). For the 3 mm diffusion model, TR-OCTA showed a BVD of 221.28, closely aligning with GT-OCTA (220.48). Similarly, BVC showed a slight increase in TR-OCTA compared to GT-OCTA. The largest deviation was observed in VPI, where TR-OCTA values were significantly lower than GT-OCTA, indicating differences in vascular perimeter index. In the 6 mm dataset, the diffusion model demonstrated larger variations, particularly in BVD (232.05 vs. 220.31) and VPI (18.72 vs. 27.18), suggesting a shift in vascular density and perimeter measurements. Comparatively, the GAN-based model had more consistent values, especially for 3 mm, with smaller deviations in BVD (210.57 vs. 206.71 for 3 mm) and BVC (22.85 vs. 22.80). Though VPI showed slight differences for 3 mm, 6 mm dataset showed a larger difference.

**Table 4:** Feature value comparison for OCT500 (3 mm & 6 mm).

OCTA dataset	FoV	BVD (Mean, SD)	BVC (Mean, SD)	BVT (Mean, SD)	VPI (Mean, SD)
BBDM					
TR-OCTA	3 mm	221.28 (12.76)	25.97 (0.9)	1.087 (0.008)	24.72 (2.11)
GT-OCTA		220.48 (12.72)	25.4 (0.5)	1.09 (0.009)	31.84 (1.87)
TR-OCTA	6 mm	232.05 (31.44)	46.20 (3.72)	1.077 (0.008)	18.72 (3.24)
GT-OCTA		220.31 (34.96)	48.27 (2.15)	1.084 (0.008)	27.18 (3.43)
GAN					
TR-OCTA	3 mm	210.57 (21.70)	22.85 (0.59)	1.088 (0.006)	30.3 (1.5)
GT-OCTA		206.71 (34.55)	22.80 (0.46)	1.088 (0.007)	31.22 (2.07)
TR-OCTA	6 mm	218.29 (34.9)	44.6 (1.72)	1.086 (0.007)	23.22 (2.96)
GT-OCTA		216.83 (34.79)	42.76 (1.55)	1.088 (0.008)	27.52 (3.4)

The UIC dataset followed similar trends, with the diffusion model exhibiting higher BVD values across all patient groups in both 3 mm and 6 mm datasets (Table 5). For the 3 mm dataset, TR-OCTA BVD values were 227.73, whereas GT-OCTA had significantly lower values at 197.49, reflecting a notable deviation. This pattern persisted in patient groups, with TR-OCTA showing consistently higher BVD values across Control, Mild, Moderate, and Severe categories. VPI values, however, were generally lower in TR-OCTA, further emphasizing differences in vascular perimeter distributions. In the 6 mm dataset, TR-OCTA BVD values remained elevated (221.73 vs. 200.45 in GT-OCTA), particularly in the Moderate (225.55 vs. 200.29) and Severe (225.81 vs. 202.27) groups. These results highlight that the diffusion model tends to overestimate BVD while maintaining comparable trends in other features, unlike the GAN-based model, which aligns more closely with GT-OCTA.

**Table 5:** Feature value comparison for UIC dataset (3 mm & 6 mm).

OCTA dataset	Dataset (no. of scans)	BVD (Mean, SD)	BVC (Mean, SD)	BVT (Mean, SD)	VPI (Mean, SD)
BBDM (3 mm)					
TR-OCTA	Complete (187)	227.73 (21.55)	26.22 (1.14)	1.081 (0.0085)	22.66 (4.11)
GT-OCTA		197.49 (25.2)	27.58 (0.87)	1.092 (0.009)	27.4 (4.29)
TR-OCTA	Control (35)	215.93 (19.16)	26.25 (0.58)	1.088 (0.009)	24.71 (2.94)
GT-OCTA		223.42 (27.64)	26.67 (0.7)	1.092 (0.008)	32.1 (4.3)
TR-OCTA	Mild NPDR (118)	230.50 (18.64)	25.96 (1.23)	1.080 (0.008)	21.89 (4.18)
GT-OCTA		189.64 (20.19)	27.84 (0.78)	1.092 (0.009)	26.01 (3.49)
TR-OCTA	Moderate NPDR (37)	225.26 (24.98)	27.04 (0.82)	1.083 (0.009)	23.19 (4.08)
GT-OCTA		197.30 (21.35)	27.55 (0.67)	1.094 (0.011)	27.59 (3.25)
TR-OCTA	Severe NPDR (97)	233.61 (21.4)	26.29 (1.25)	1.081 (0.008)	22.02 (4.19)
GT-OCTA		189.68 (21.31)	27.63 (0.69)	1.091 (0.009)	25.89 (3.59)
GAN (3 mm)					
TR-OCTA	Complete (187)	220.91 (21.73)	18.21 (0.86)	1.078 (0.0066)	24.52 (2.70)
GT-OCTA		189.29 (25.59)	20.95 (0.65)	1.090 (0.0086)	26.11 (4.45)
TR-OCTA	Control (35)	212.69 (17.66)	18.38 (0.80)	1.083 (0.0082)	25.48 (1.90)
GT-OCTA		217.57 (27.82)	20.25 (0.48)	1.089 (0.0073)	31.30 (4.53)
TR-OCTA	Mild NPDR (118)	223.29 (22.97)	18.00 (0.86)	1.078 (0.0056)	23.99 (2.76)
GT-OCTA		180.42 (19.80)	21.17 (0.58)	1.091 (0.0088)	24.56 (3.47)
TR-OCTA	Moderate NPDR (37)	216.40 (24.49)	18.51 (0.97)	1.078 (0.0065)	24.73 (2.45)
GT-OCTA		189.60 (20.77)	20.91 (0.47)	1.091 (0.0102)	26.29 (3.25)
TR-OCTA	Severe NPDR (97)	229.30 (18.85)	17.96 (0.95)	1.077 (0.0061)	23.83 (2.76)
GT-OCTA		181.44 (20.79)	20.99 (0.50)	1.090 (0.0087)	24.51 (3.57)
BBDM (6 mm)					
TR-OCTA	Complete (258)	221.73 (34.84)	47.1 (3.31)	1.078 (0.007)	20.01 (3)
GT-OCTA		200.45 (13.35)	52.67 (1.089)	1.089 (0.007)	27.7 (2.25)
TR-OCTA	Control (59)	219.73 (27.96)	47.79 (2.59)	1.078 (0.008)	21.51 (2.41)
GT-OCTA		197.56 (11.21)	52.41 (0.90)	1.089 (0.007)	27.63 (1.78)
TR-OCTA	Mild NPDR (143)	225.55 (33)	46.43 (3.37)	1.077 (0.007)	19.51 (2.85)
GT-OCTA		200.29 (12.2)	52.77 (1.05)	1.088 (0.007)	27.43 (2.08)
TR-OCTA	Moderate NPDR (69)	212.89 (41.77)	48.02 (3.46)	1.079 (0.007)	20.38 (3.18)
GT-OCTA		204.31 (13.25)	52.52 (1.16)	1.089 (0.006)	28.34 (2.16)
TR-OCTA	Severe NPDR (123)	225.81 (38.49)	46.68 (3.49)	1.077 (0.007)	19.27 (3.04)
GT-OCTA		202.27 (13.16)	52.52 (1)	1.089 (0.008)	27.41 (2.34)
GAN (6 mm)					
TR-OCTA	Complete (258)	214.75 (25.5)	37.30 (0.93)	1.084 (0.0059)	25.66 (2.86)
GT-OCTA		199.76 (15.05)	39.54 (0.87)	1.089 (0.0069)	27.14 (2.46)
TR-OCTA	Control (59)	211.57 (21.79)	37.35 (0.72)	1.085 (0.0064)	28.55 (2.19)
GT-OCTA		197.61 (12.83)	39.40 (0.74)	1.088 (0.0062)	27.43 (2.027)
TR-OCTA	Mild NPDR (143)	218.19 (22.93)	37.12 (1.067)	1.084 (0.0056)	25.86 (2.74)
GT-OCTA		199.29 (14.16)	39.60 (0.80)	1.088 (0.0070)	26.76 (2.30)
TR-OCTA	Moderate NPDR (69)	209.54 (29.57)	37.64 (0.83)	1.084 (0.0060)	27.00 (2.42)
GT-OCTA		204.29 (14.62)	39.41 (0.93)	1.089 (0.0072)	27.81 (2.31)
TR-OCTA	Severe NPDR (123)	216.41 (25.25)	37.14 (0.81)	1.083 (0.0057)	26.12 (2.44)
GT-OCTA		201.34 (14.96)	39.34 (0.84)	1.089 (0.0072)	26.72 (2.52)

**Figure 3:**
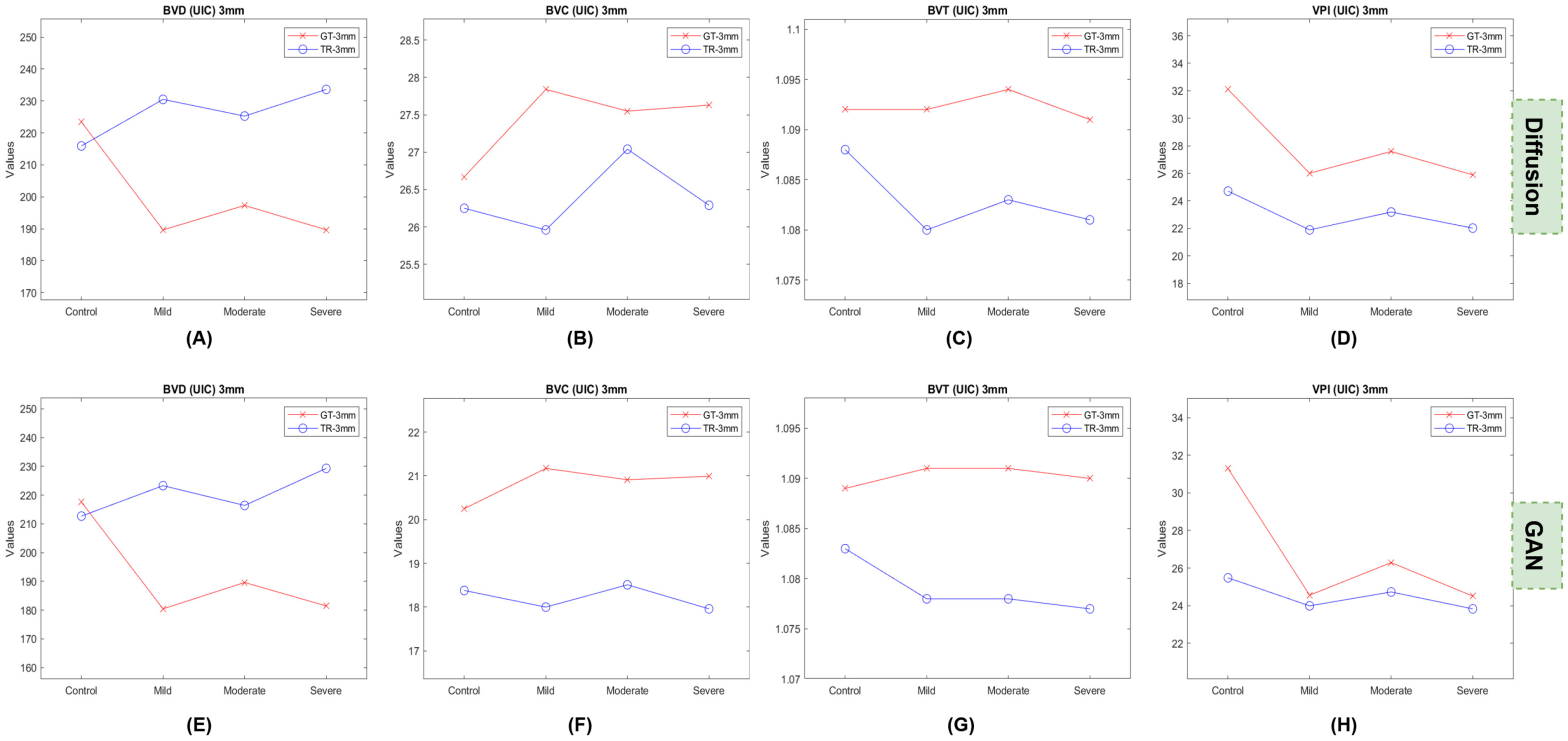
Feature plots for UIC 3 mm dataset. **(A,E)** show BVD feature values, **(B,F)** BVC, **(C,G)** BVT, **(D,H)** VPI.

**Figure 4:**
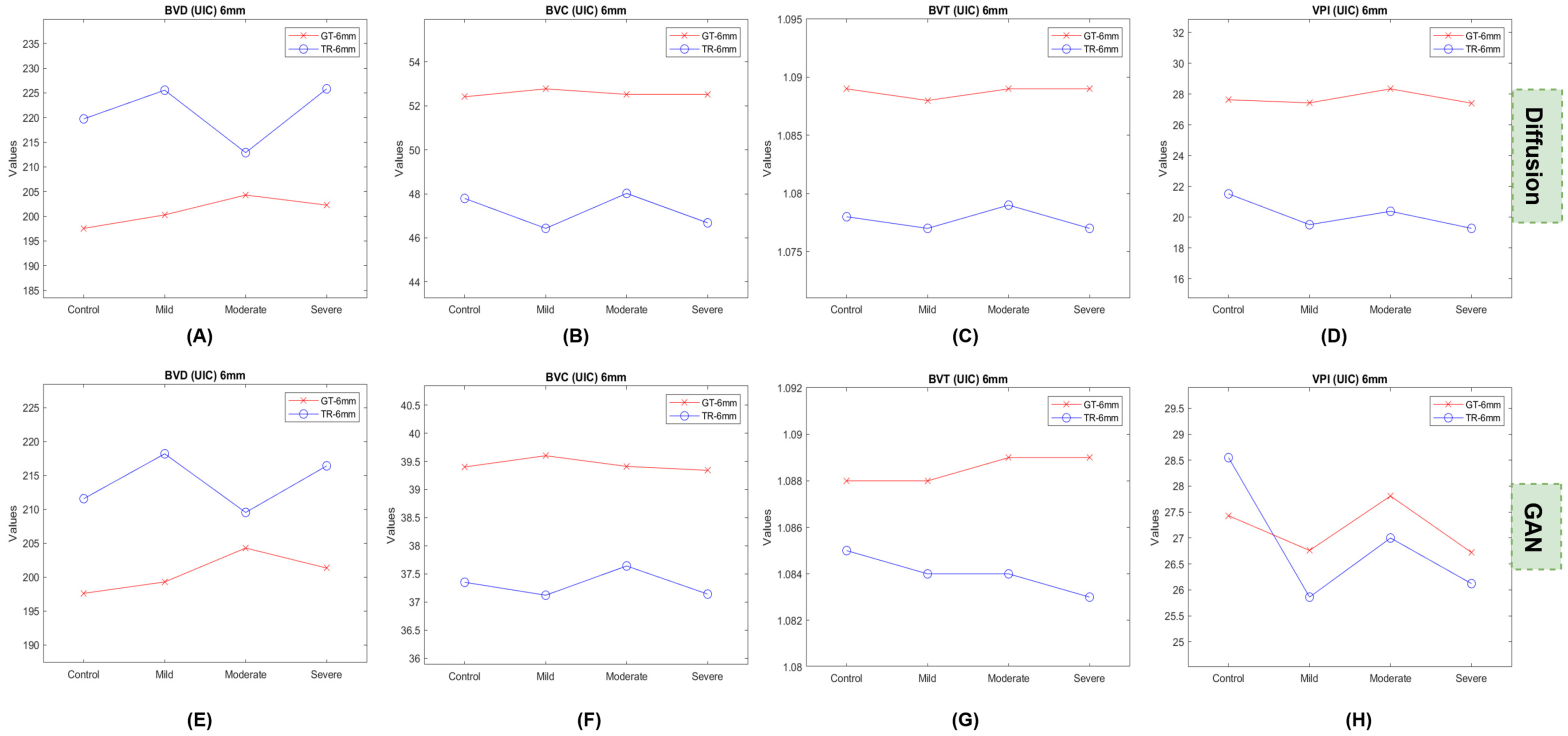
Feature plots for UIC 6 mm dataset. **(A,E)** show BVD feature values, **(B,F)** BVC, **(C,G)** BVT, **(D,H)** VPI.

We also plotted the feature values for the UIC dataset categorically to observe if the values show any trend similar to the GAN model generated TR-OCTA. Figures 3, 4 represent the UIC 3 mm and 6 mm data values, respectively. It is clear from the feature plots (Figure 3) that despite the wider differences from the GT values the overall trend among different patient groups is similar between the two models. The trend is prominent for VPI in both model and moderate NPDR patients can be distinguished easily from the plots. When observed Figure 4 we notice a similar pattern to the GAN model except for VPI. For all categories of patients TR-OCTA almost follow the pattern set by the GT-OCTA except for BVD.

Ablation study we also performed ablative experiments to verify the effectiveness of Brownian Bridge condition on the diffusion model by comparing OCTA projection quality against a standard CDM (Supplementary Figures 1, 2). As part of our ablation study, we implemented a conditional Denoising Diffusion Probabilistic Model (DDPM) to translate OCT to OCTA images. The model conditions on structural OCT input to generate vascular OCTA outputs by employing a U-Net architecture augmented with sinusoidal time-step embeddings. During training, noise is added to OCTA images across a fixed diffusion schedule, and the model learns to reconstruct clean images conditioned on corresponding OCT slices. We used an L1 loss on predicted clean images rather than denoising noise directly, allowing more faithful reconstructions. For efficient inference, DDIM sampling was adopted to accelerate generation. This model serves as a diffusion-based baseline for evaluating translation fidelity against our proposed BBDM framework. We trained both BBDM and CDDPM for 10 epochs and tested on some random UIC patients to compare vascular structure quality.

## 4 Discussion

In this study, we demonstrated a diffusion-based generative AI approach for OCT-to-OCTA translation. We implemented the BBDM (43) model as an image-to-image translation for OCTA images and compared the results with our previous study of a GAN based image-to-image translation model to verify the use of a diffusion model for retinal image translation task. We trained the BBDM on OCT500 and tested on UIC dataset, both having two FoVs: 3 mm and 6 mm. Due to the model’s internal architecture it learns to translate by adding and removing noise, fundamentally different than GAN based model that used contextual and semantic information. The BBDM was selected in this study for OCT to OCTA translation due to its ability to effectively capture modality specific distributions while simplifying the training pipeline. Unlike traditional diffusion models that use a unidirectional forward-noise and reverse-denoise process, BBDM introduces a bidirectional, condition aware generation pathway. This design bridges the start and end modalities OCT and OCTA via a Brownian bridge process, allowing the model to learn the translation distribution directly without requiring intermediate denoising or auxiliary conditioning steps. This makes BBDM more stable and computationally efficient compared to classical DDPMs and avoids the architectural complexity of classifier guidance or score distillation methods.

When compared to GANs, BBDM demonstrates several critical advantages. GANs, while achieving slightly lower FID scores due to their fine grained generative ability, often struggle with training instability, mode collapse and generalization to unseen data. In contrast, BBDM consistently outperformed GANs in preserving perceptual and structural image quality across various scan types, as reflected in higher PCQI scores and competitive SSIM values, especially for wider FoV images. More importantly, BBDM showed superior consistency in generating clinically meaningful vascular features such as BVC, BVT, and VPI, across both seen and unseen datasets. These features are often distorted or underrepresented in GAN-generated outputs due to adversarial overfitting. Thus, BBDM not only improves translation fidelity but also enhances downstream clinical utility. We therefore recommend BBDM as a more robust, generalizable, and diagnostically faithful approach for modality translation tasks in retinal imaging.

We performed a comprehensive analysis of the TR-OCTA images in terms of quality (SSIM, FID, PCQI) and quantitative parameters (BVD, BVC, BVT, VPI) to validate the use of a condition diffusion model and compared the performance based on these parameters against a GAN based model. From the analysis we found that FID and PCQI scores, overall, were better than the GAN model suggesting better structural representation captured by the diffusion model. Despite the significant differences, feature plots provide reliable information that a combination of BVC, BVT and VPI features can be considered for distinguishing DR patients.

The quantitative analysis using two-tailed t-test (*p* < 0.05) highlights significant deviations in vascular feature distributions for the diffusion model, particularly when applied to the unseen UIC dataset (Table 1). Since the model was trained on the OCT500 dataset, its performance on OCT500 reflects its ability to replicate learned vascular distributions, whereas its performance on UIC serves as an indicator of its generalization capability. In the OCT500 3 mm dataset, the diffusion model exhibited moderate deviations in BVD but BVC, BVT and VPI showed statistically significant differences, suggesting structural inconsistencies in TR-OCTA images. The discrepancies became more pronounced in the 6 mm dataset, where all the feature values showed variations emphasizing systematic shifts in vascular topology as the field of view increased. When tested on the unseen UIC dataset, all the features exhibited statistical deviations. In the 3 mm, BVD, BVC, BVT and VPI followed the same trend as in the OCT500 dataset highlighting the difference between generated and true values being statistically significant, meaning the model did not accurately replicate that feature’s distribution. The 6 mm scans show similar significant deviation. In comparison, the GAN-based model exhibited lower deviations in some cases but still demonstrated statistically significant differences across features. The diffusion model, although generally more perceptually consistent, struggles to preserve fine vascular attributes statistically across datasets similar to the GAN model. Hence although BBDM performs slightly worse than GAN, its overall performance is similar to GAN making it a potential alternative.

SSIM (Table 2) results indicate that the diffusion model maintains higher perceptual quality in larger FoVs. For the OCT500 3 mm dataset, BBDM achieves slightly lower yet closer SSIM value than the GAN model however it achieved a higher SSIM in OCT500 6 mm, compared to the GAN model, suggesting that the stochastic bridging process in BBDM facilitates smoother image transitions and enhanced structural integrity. As for an independent dataset, UIC, the diffusion model demonstrated slightly improved SSIM scores compared to the GAN model. For UIC 3 mm, BBDM surpasses the GAN with a small margin and slightly larger fluctuation across disease groups. This behavior reflects the UIC dataset’s higher variability and mixed disease stages, where BBDM’s whole-image modeling can be more sensitive to heterogeneous input quality. Importantly, these differences diminish and even reverse as the field of view increases-BBDM consistently achieves higher SSIM in the 6 mm scans for both OCT500 and UIC, reinforcing its capacity to preserve critical image structures even in more complex real world datasets. This is also due to the reason that the GAN model focused only on the vasculature rather than the whole image therefore SSIM values are lower compared to the diffusion model. Therefore, SSIM is an ideal metric BBDM generated TR-OCTA. Another critical issue is the GAN’s focus to segment the vasculature and leverage the information for fine detail. However, the data itself does not show the deep layer vasculature properly making it difficult to perform vascular segmentation which is one of the important modules of the GAN. On the other hand, BBDM focuses on the whole image without prioritizing any specific feature or vasculature hence shows better structural similarities even on the unseen UIC dataset making it a better choice for structural generalizability. This is also evident from the Table 2 SSIM values showing clear distinction among control and other DR stage patients generated by BBDM whereas GAN performs a bit random for the unseen UIC dataset specially for UIC 6 mm patients.

To further assess the generative quality of the BBDM and GAN model, we evaluated FID and PCQI scores (Table 3). FID measures image fidelity by comparing the statistical distributions of generated and real images, with lower scores indicating higher fidelity, whereas PCQI quantifies perceptual quality, with values closer to 1 reflecting better visual coherence. A key observation is that the diffusion model consistently exhibited higher FID scores across all datasets and scan sizes, indicating a greater deviation from real OCTA image distributions compared to the GAN model suggesting that its generated images are less realistic and deviated more from the statistical properties of real OCTA images. However, it is important to note that the FID score improved significantly at 6 mm, aligning with the higher SSIM scores observed for 6 mm scans in Table 2. This reinforces the idea that the diffusion model’s stochastic bridging mechanism is more effective at maintaining global coherence in larger FoV.

When tested on the more complex UIC dataset, the diffusion model exhibited even higher FID scores indicating a greater struggle in adapting to generalized datasets. This can be attributed to dataset’s inherent variability in acquisition quality and overlapping patient records where disease severity progressed over time. Moreover, the absence of a feedback based architectural unit, e.g., an adversarial discriminator, which prevents it from refining generated images to closely match real clinical OCTA scans. Unlike the GAN-based model, which continuously improves image realism through generator-discriminator feedback loops, the diffusion model relies purely on iterative noise removal without direct supervision from real image distributions. This likely explains why its FID scores remain significantly higher, especially in the UIC dataset, where patient-specific variations and disease severity introduce greater image diversity.

Despite the lower image fidelity indicated by FID, the diffusion model consistently achieved high PCQI scores across both datasets and both FoVs. These results suggest that, even though the diffusion model struggles with statistical alignment to real OCTA images, it generates perceptually convincing outputs that maintain structural coherence. One possible explanation for the high PCQI scores is the Brownian Bridge formulation of the diffusion model, which ensures a smooth and gradual transition from the source (OCT) to target (OCTA) latent representations. Unlike traditional diffusion models that introduce excessive noise at each step, BBDM carefully balances variance scheduling, enabling more stable intermediate states and perceptually appealing reconstructions. This is further reflected in the higher PCQI scores at 6 mm, where the model benefits from its structured approach to noise removal and latent space interpolation.

In terms of hallucination quality, the BBDM generated TR-OCTA images achieved significantly lower hallucination scores compared to those produced by GAN based model across a sample of 500 TR-OCTA images, demonstrating the superior reliability and visual fidelity of the diffusion model for clinically relevant image translation. This reduction in hallucinated vascular structures is particularly important, as false vessel patterns can mislead clinical interpretation and undermine trust in automated OCTA generation. By quantitatively assessing and minimizing such artifacts through a structured hallucination quality score, our approach provides a more dependable framework for translating OCT to OCTA with improved diagnostic consistency.

From the ablation study, compared against CDDPM, we verified that implementation of Brownian Bridge improves the vascular structural integrity. BBDM conditions noise addition based on target OCTA rather than simply taking OCT as a conditional input. This can also be contributed to the CDDPM’s inability to generalize during validation as evident by Supplementary figures.

For quantitative evaluation, four vascular biomarkers: BVD, BVC, BVT, and VPI were computed for both datasets and compared with the GAN model to assess the performance of the BBDM. Results from the OCT500 dataset (Table 4) indicate that BBDM consistently overestimates BVD and BVC for both 3 mm and 6 mm scans relative to GT-OCTA, implying that the diffusion model generates a denser vascular network, possibly due to stochastic bridging effects in its latent space. Similar trends are observed in the GAN model but with smaller deviations from the ground truth. Both models perform comparably in BVT, showing minimal deviation from GT-OCTA, likely because vessel tortuosity is a stable anatomical feature less influenced by adversarial or stochastic training dynamics. However, BBDM exhibits larger discrepancies in VPI, suggesting that its iterative denoising process overly smooths vessel boundaries and removes fine vascular edges. In contrast, the GAN model achieves VPI values closer to GT-OCTA, as its adversarial training better preserves sharp boundary structures and contextual vascular details that the diffusion model lacks.

For the UIC dataset BBDM exhibits a similar scenario of higher BVD values compared to the GT-OCTA following the same tendency as the GAN model but with higher discrepancies. For BVC however, the DM produced TR images with values generally closer to GT-OCTA, while the GAN model consistently underestimated BVC across all groups. Similar to OCT dataset, BVT values were better preserved for both models with GAN having slightly better representation of GT images. Unlike BVD, VPI values were observed to be lower across all categories particularly in the severe group. In contrast, GAN model was able to preserve the characteristics of GT-OCTA better with smaller variation. From the feature plots for UIC 3 mm (Figure 3) an inconsistent pattern is observed for BVD from the TR-OCTA for both models having a larger variation for mild stage patients. BVC shows a better trend representation, especially for moderate stage of DR, which continues for other features BVT and VPI. For both models, VPI here can be potentially considered as an important feature to identify DR patients clearly distinguishing different stages of severity. For BVC and BVT, DM shows better discrepancies in terms of values among patients compared to the GAN suggesting a combination of BVC, BVT and VPI can be a potential biomarker for DR patients. On the other hand, from Figure 4, we observe the DM having a smoother trend compared to 3 mm but following a similar pattern set by GAN generated TR-OCTA for BVC and BVT. For VPI, GAN shows a better distinction between the GT and TR-OCTA solidifying our suggestion of using VPI as a potential biomarker. Considering larger FoV, DM generated feature values maintain an almost identical shape while distinguishing moderate stage DR patients in all scenarios except for BVD.

In this study, our external validation is based on a single clinical dataset (UIC) however the design of our study intentionally incorporates diversity in both imaging conditions and population characteristics to assess generalizability. The primary dataset, OCT500, is a large-scale, public dataset from Jiangsu Province, China, comprising 500 subjects with a wide range of disease diversity. In contrast, the UIC dataset, collected from Chicago (Cook County) consists of retrospectively collected OCT and OCTA scans from DR patients (Type II) imaged in a real-world clinical setting at the University of Illinois at Chicago. This dataset includes patients stratified by NPDR severity (mild, moderate, and severe). Both datasets were collected using different devices and they represent different demographic patients. Visually, UIC dataset has varying quality images and different histograms than OCT500 making it a proper independent test set mimicking real life clinical scenario. These differences in data source, imaging protocol, disease distribution, and clinical setting collectively represent a robust test of the model’s ability to generalize beyond the original training domain. We also applied domain adaptation (Supplementary Table 1) to the UIC test set to mimic OCT500 dataset style and got expected results. For vascular features, especially, the model generates better quality OCTA overall. The difference between GT and TR-OCTA reduces as domain adaptation helps to reduce style differences between the datasets by improving overall performance.

Overall, we can safely deduce that BBDM, as a conditional diffusion model, is a viable option for modality translation even at this early stage and without task-specific modifications. While GAN-based models often produce visually sharper OCTA images, such apparent detail may stem from hallucinated features introduced during adversarial training, rather than true vascular anatomy. By contrast, the higher SSIM values achieved by BBDM demonstrate that its architecture focuses on preserving global structural representation rather than emphasizing superficial sharpness. Although FID scores remain relatively high, the consistently strong PCQI scores indicate that BBDM effectively maintains perceptual contrast and overall image coherence, which are more relevant for clinical interpretability. With respect to quantitative vascular features, we acknowledge that TR-OCTA values do not perfectly match GT-OCTA; however, our analysis shows that BBDM reliably preserves the relative progression trends across DR stages, such as the expected decline in vascular perimeter and complexity from control to severe DR. This trend of fidelity is of greater clinical value than exact numerical alignment, given that TR-OCTA is generated from structural OCT rather than direct flow data. Moreover, compared to GANs, BBDM avoids hallucinated vessels and ensures structural robustness and reproducibility, which are essential for reliable biomarker extraction. From our vascular feature analysis, we highlight that BVC, BVT, and VPI remain reliable markers for distinguishing DR severity across datasets, while BVD exhibits greater variability. These clarifications underscore that BBDM prioritizes trustworthy structural fidelity and clinically meaningful feature trends over potentially misleading sharpness, making it a robust and generalizable alternative to GAN-based approaches.

Despite the BBDMs limitations in terms of quality and quantitative feature representation compared to the GAN, the DM performed considerably well considering it relies solely on learned noise transitions to generate OCTA structures unlike the GAN model which actively refines vascular structures based on real image comparisons contributing to a more controlled generation of TR-OCTA. Besides, our study has other limitations inherent to the procedure. One major limitation is the scarcity of publicly available data containing paired OCT, OCTA images severely restricting the research opportunity. Additionally, the dataset used in this study is relatively small, particularly for cross-pathological investigations, limiting the generalizability of the findings. The unequal distribution of data across different patient categories and pathologies further poses a challenge, potentially introducing bias and affecting the robustness of our conclusions. Another significant issue is the inconsistency in image quality and signal strength, as not all OCTA images exhibit uniform signal integrity, which can negatively impact the accuracy of translated OCTA in both quantitative and qualitative assessments. Moreover, OCT500 and UIC datasets are collected using different devices which can also affect the source material leading to subtle structural differences. Although our dataset contains an unequal distribution across control and DR stages for UIC, we did not employ stratified sampling or weighted loss in our current framework. Instead, we ensured robustness through the use of a fixed training-validation-test split (70–5–25%) and by validating the model’s performance across multiple subsets with varying pathological distributions. The use of multiple quantitative and perceptual metrics further supports the consistency of our model’s performance across different disease severities. To our knowledge, this is the first study of diffusion model implementation to translate OCTA from OCT and extensive analysis of retinal features from the TR-OCTA images. Here we introduce DM as an alternative option to GAN, albeit showing greater variations in feature values comparably, which emphasizes the possibility of exploring other methods to find the best translation algorithm eventually.

To conclude, this study represents a significant advancement in the field of ophthalmic image translation by demonstrating the potential of DM for OCT to OCTA conversion. By leveraging a conditional DM, we introduced and validated a novel approach that eliminates the dependency on adversarial training, ensuring smoother and more structurally coherent image translations. While the GAN based model performs slightly better albeit only in feature level accuracy, BBDM presents notable advantages that make it a compelling alternative for OCT-to-OCTA translation. Unlike GAN, BBDM is architecturally simpler and avoids adversarial training, which eliminates challenges such as training instability and mode collapse. BBDM’s bidirectional stochastic approach allows for a smoother and more globally consistent translation, especially effective in wider FoV scans like 6 mm, where it outperforms GANs in structural similarity and perceptual quality. Furthermore, BBDM integrates an accelerated sampling process that significantly reduces computational overhead by sampling only key steps along the noise removal trajectory, offering faster inference without compromising image quality. This contrasts with GAN, which depends heavily on discriminative feedback, resulting in longer and potentially less stable training cycles. Another key advantage is reduced hallucination: while GANs tend to introduce fine details at the cost of generating unrealistic vascular features (due to overfitting or discriminator biases), BBDM produces more coherent and structurally plausible vascular maps. Although the diffusion model slightly underperforms in preserving exact quantitative vascular features like BVD or VPI on unseen datasets, it maintains the overall anatomical layout with greater reliability, a crucial trait for clinical interpretation. Given these strengths: better global coherence, lower risk of artifacts, faster and simpler implementation and comparable clinical feature trends, BBDM is recommended as a robust and generalizable solution for modality translation tasks in medical imaging. Its performance, particularly in structural fidelity and perceptual realism, supports its future adoption and refinement in clinical workflows.

The ability to generate high-quality OCTA images from standard OCT scans has the potential to revolutionize retinal diagnostics, reducing reliance on expensive OCTA hardware and expanding access to critical vascular imaging for patients worldwide. This study lays the groundwork for further improvements in DM based medical image translation, paving the way for enhanced deep-learning-driven ophthalmic diagnostics and broader clinical applications.

## Data Availability

Publicly available datasets were analyzed in this study. This data can be found at: OCT500: https://ieee-dataport.org/open-access/octa-500UIC: available on request.

## Footnotes

1
https://doi.org/10.1093/bjrai/ubae013

2 DOI: 10.1109/JBHI.2024.3385504.

3 Code can be found at: https://github.com/xuekt98/BBDM.
