# Supplementary material for: Diffusion model based OCT to OCTA translation
Source: Front Med (Lausanne). 2025 Nov 28;12:1655453. doi: 10.3389/fmed.2025.1655453 (PMC12698582; doi:10.3389/fmed.2025.1655453)
Supplement: Supplementary file 2 [file Image_2.pdf]

Supplementary Material

1 Supplementary Figures

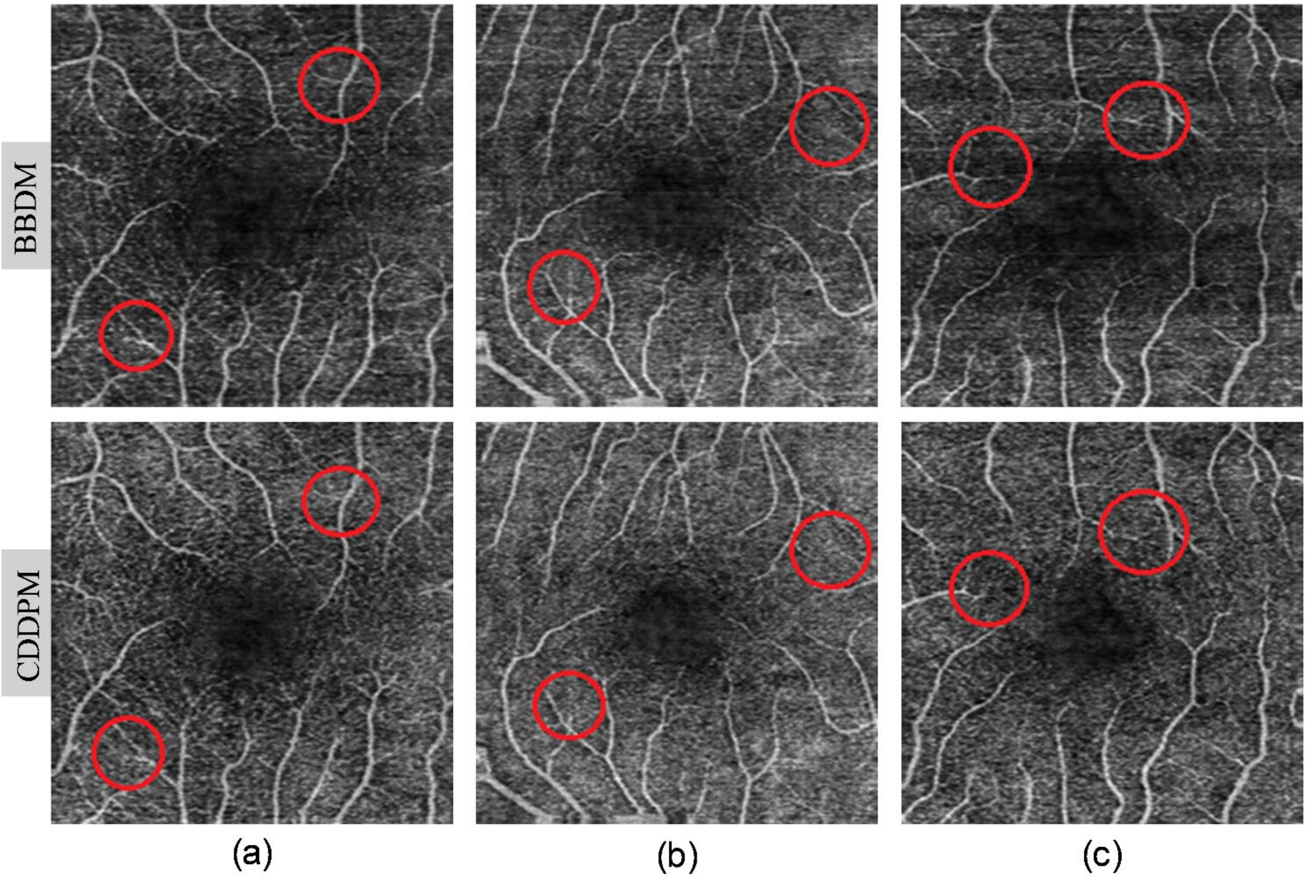

Supplementary Figure 2. Vascular structural integrity comparison between BBDM and CDDPM.
