# Supplementary material for: Diffusion model based OCT to OCTA translation
Source: Front Med (Lausanne). 2025 Nov 28;12:1655453. doi: 10.3389/fmed.2025.1655453 (PMC12698582; doi:10.3389/fmed.2025.1655453)
Supplement: Supplementary file 3 [file Table_1.docx]

Supplementary Material

# Supplementary Table 1

| **FID** | **Diffusion** | **Diffusion (domain adapted)** |
| --- | --- | --- |
| UIC 3mm | 222.71 | 236.37 |
| UIC 6mm | 153.27 | 176.56 |

| **PCQI** | **Diffusion** | **Diffusion (domain adapted)** |
| --- | --- | --- |
| UIC 3mm | 0.9952±.0008 | 0.99503 (.000775) |
| UIC 6mm | 0.9959±.00053 | 0.99568 (0.000532) |

| **3mm** | **GT OCTA** | **Diffusion**  **(TR OCTA)** | **Diffusion (domain adapted)**  **(TR OCTA)** |
| --- | --- | --- | --- |
| BVD | 197.49 (25.2) | 227.73 (21.55) | 222.81(17.60) |
| BVC | 27.58 (0.87) | 26.22 (1.14) | 25.97(0.84) |
| BVT | 1.092 (0.009) | 1.081 (0.0085) | 1.084 (.0077) |
| VPI | 27.4 (4.29) | 22.66 (4.11) | 26.84(2.76) |

| **6mm** | **GT OCTA** | **Diffusion**  **(TR OCTA)** | **Diffusion (domain adapted)**  **(TR OCTA)** |
| --- | --- | --- | --- |
| BVD | 200.45 (13.35) | 221.73 (34.84) | 217.37(25.87) |
| BVC | 52.67 (1.089) | 47.1 (3.31) | 46.77(3.18) |
| BVT | 1.089 (0.007) | 1.078 (0.007) | 1.081(.0076) |
| VPI | 27.7 (2.25) | 20.01 (3) | 21.83(2.52) |
