# Supplementary material for: Diffusion model based OCT to OCTA translation
Source: Front Med (Lausanne). 2025 Nov 28;12:1655453. doi: 10.3389/fmed.2025.1655453 (PMC12698582; doi:10.3389/fmed.2025.1655453)
Supplement: Supplementary file 4 [file Data_Sheet_1.docx]

Supplementary Material

# Materials and methods

**Qualitative Metrics**

**Hallucination quality score** Based on the visible vascular hallucination, a scoring system (0-10) was implemented by an expert clinician. Lower score means lower fake or hallucinated vascular structures and higher score indicating higher hallucination.

**SSIM** Structural Similarity Index Measure (SSIM) is a widely used metric for evaluating the similarity between two images by mimicking human visual perception. Unlike traditional measures like Mean Squared Error (MSE) or Peak Signal-to-Noise Ratio (PSNR), SSIM considers structural information, luminance and contrast to provide a more perceptually relevant assessment.

**FID** Frechet Inception Distance (FID) score is another widely used metric for evaluating the quality of generated images by comparing their distribution to that of real images. It measures the similarity between feature representations of real and generated images using the activations of a pre-trained neural network, typically Inception-v3. Unlike pixel-wise metrics, FID considers higher-level semantic features and captures perceptual differences effectively. A lower FID score indicates better quality and higher realism in generated images.

**PCQI** Perception-based Contrast Quality Index (PCQI) is a metric designed to evaluate image quality by focusing on perceptual contrast. Unlike traditional metrics, PCQI models the human visual system’s sensitivity to contrast changes and evaluates quality based on local contrast distortions. It considers multi-scale contrast comparisons across different image regions, making it effective for assessing contrast variations due to compression, noise, or other degradations. PCQI is particularly useful in applications where maintaining perceptual contrast fidelity is critical, such as medical imaging and image enhancement.

**Quantitative Metrics**

**BVD** Blood vessel density or vessel density (48) is a quantitative OCTA derived metric defined as the ratio of blood vessel area to the total imaged area, offering an objective indicator of retinal vascular health. BVD has been effectively utilized to identify early signs of retinal pathologies such as DR and AMD (49,50). In OCTA imaging, BVD quantifies the extent of perfused capillaries and is sensitive to local vascular changes, making it a valuable parameter for detecting subtle microvascular abnormalities.

**BVC** Blood vessel caliber, another feature used for this study, calculates the ratio of vessel area to the vessel length (29) and is a critical quantitative vascular biomarker. It is typically assessed by identifying vessel centerlines and measuring cross-sectional widths on enface or phase resolved OCT/OCTA images. In ophthalmic imaging, BVC provides insight into vascular adaptations or pathologies: for example, arterial narrowing may indicate systemic hypertension, while venous dilation could suggest retinal ischemia or compromised perfusion (51). Accurate quantification of vessel caliber on OCTA supports evaluation of physiological and pathological hemodynamic changes, making BVC a valuable parameter for early detection and monitoring of retinal and systemic vascular diseases.

**BVT** It (18) quantifies the degree of bending and twisting along a vessel’s path, serving as a key biomarker for vascular abnormalities in both ocular and systemic diseases. BVT can be described as the integration of several geometric features: the variability in vessel direction along its length (measured by standard deviation of tangent angles), the count of points where the vessel changes curvature direction (inflection points), the actual length of the vessel (arc length) and the straight-line distance between its endpoints (chord length). Its application in OCTA has reliably detected elevated tortuosity in conditions like sickle cell retinopathy and Fabry disease, confirming BVT as a sensitive and reproducible marker of microvascular remodeling (52,53).

**VPI** VPI (50) quantifies the total length of vessel boundaries normalized to the image area, providing a sensitive measure of vascular boundary complexity beyond mere vessel presence. It is calculated by dividing the sum of the perimeters of all detected vessel segments (i.e., the contour pixel length) by the total imaged area. VPI has been shown to significantly increase in retinal conditions characterized by microvascular remodeling such as non‑proliferative DR and sickle cell retinopathy (SCR) where capillary dilation and branching complexity produce longer vessel boundaries relative to imaged area.

To draw the comparison between GAN model and DM we also performed the same statistical analysis based on these features to quantify the TR OCTA. This will provide a clear distinction for the DM as a translation algorithm for medical imaging.

Supplementary material is not typeset so please ensure that all information is clearly presented, the appropriate caption is included in the file and not in the manuscript, and that the style conforms to the rest of the article.
